# Microglia activation and neuronal alterations in retinas from COVID-19 patients: correlation with clinical parameters

**DOI:** 10.1186/s40662-023-00329-2

**Published:** 2023-03-01

**Authors:** Henar Albertos-Arranz, Natalia Martínez-Gil, Xavier Sánchez-Sáez, Agustina Noailles, Clara Monferrer Adsuara, Lidia Remolí Sargues, Juan J. Pérez-Santonja, Pedro Lax, Ramón Calvo Andrés, Nicolás Cuenca

**Affiliations:** 1 Department of Physiology, Genetics and Microbiology, University of Alicante, San Vicente del Raspeig Road W/N, 03690 Alicante, Spain; 2 Department of Ophthalmology, General University Hospital Consortium of Valencia (CHGUV), 46014 Valencia, Spain; 3 Department of Ophthalmology, General University Hospital of Alicante (HGUA), 03010 Alicante, Spain; 4 Alicante Institute for Health and Biomedical Research (ISABIAL), Alicante, Spain; 5 National Institute of Health Carlos III (ISCIII), (RETICS) Cooperative Health Network for Research in Ophthalmology (Oftared), 28040 Madrid, Spain

**Keywords:** Human eyes, Retina, SARS-CoV-2, Microglia, Gliosis, Müller cells, ACE2

## Abstract

**Background:**

Different ocular alterations have been described in patients with coronavirus disease 2019 (COVID-19). Our aim was to determine whether COVID-19 affected retinal cells and establish correlations with clinical parameters.

**Methods:**

Retinal sections and flat-mount retinas from human donors with COVID-19 (n = 16) and controls (n = 15) were immunostained. The location of angiotensin-converting enzyme 2 (ACE2) and the morphology of microglial cells, Müller cells, astrocytes, and photoreceptors were analyzed by confocal microscopy. Microglial quantification and the area occupied by them were measured. Correlations among retinal and clinical parameters were calculated.

**Results:**

ACE2 was mainly located in the Müller cells, outer segment of cones and retinal pigment epithelium. Cell bodies of Müller cells in COVID-19 group showed greater staining of ACE2 and cellular retinaldehyde-binding protein (CRALBP). The 81.3% of COVID-19 patients presented disorganization of honeycomb-like pattern formed by Müller cells. Gliosis was detected in 56.3% of COVID-19 patients compared to controls (40%) as well as epiretinal membranes (ERMs) or astrocytes protruding (50%). Activated or ameboid-shape microglia was the main sign in the COVID-19 group (93.8%). Microglial migration towards the vessels was greater in the COVID-19 retinas (*P* < 0.05) and the area occupied by microglia was also reduced (*P* < 0.01) compared to control group. Cone degeneration was more severe in the COVID-19 group. Duration of the disease, age and respiratory failure were the most relevant clinical data in relation with retinal degeneration.

**Conclusions:**

The retinas of patients with COVID-19 exhibit glial activation and neuronal alterations, mostly related to the inflammation, hypoxic conditions, and age.

**Supplementary Information:**

The online version contains supplementary material available at 10.1186/s40662-023-00329-2.

## Background

Severe acute respiratory syndrome coronavirus 2 (SARS-CoV-2) emerged in Wuhan, China, in December 2019, causing the coronavirus disease 2019 (COVID-19 [1]. Since then, the spread of SARS-CoV-2 has triggered a pandemic which was declared by World Health Organization (WHO) on 11 March 2020. As of 26^th^ August 2022, more than 596 million COVID-19 cases have been reported worldwide causing about 6.5 million deaths worldwide [2].

Although the principal symptoms of COVID-19 are related to respiratory infection [3], clinical manifestations also involve other tissues and organs [4, 5]. In fact, neuropathological signs associated with COVID-19 have been described [6, 7]. Specifically, inflammation was found in the postmortem brains of COVID-19 patients even though the SARS-CoV-2 was not detected in all of them [6, 8]. Moreover, the presence of SARS-CoV-2 or a greater disease severity was not associated with increased glial activation [6].

Several articles have identified the presence of the SARS-CoV-2 in the retina of some patients through reverse-transcription polymerase chain reaction (RT-PCR) and immunohistochemistry [9, 10]. Different ocular manifestations could be also related to COVID-19, such as conjunctivitis, dry eye, or foreign body sensation and ocular pain [11, 12]. In fact, some authors hypothesize the possible SARS-CoV-2 entrance to the respiratory system through the ocular surface since the cornea and conjunctiva present angiotensin-converting enzyme 2 (ACE2) receptors [13, 14]. Otherwise, although different studies have observed alterations in the conjunctiva [13], choroid or retinal vasculature [15] of COVID-19 patients; to the best of our knowledge, our study has the largest number of samples which classifies the response to damage of different retinal cell types associated to this disease. In addition, this is the first study to correlate retinal degeneration at the cellular level with clinical signs. An in-depth immunohistochemical study was performed to determine whether COVID-19 affects retinal cells, and clinical correlations established.

## Methods

### Study design

A cross-sectional, comparative study of the retinal histology between patients deceased by COVID-19 and donors deceased by causes unrelated to SARS-CoV-2 was performed. The inclusion criteria for the study group were a COVID-19 diagnosis confirmed by quantitative RT-PCR analysis upon admission to hospital, and a good general condition of the samples before and after ocular dissection. Inclusion criteria in the control group comprised absence of retinal alterations and other systemic diseases which could clearly affect the retina.

### Source and management of human donor eyes

Human donor eyes with COVID-19 (n = 16) were analyzed postmortem from the General University Hospital Consortium of Valencia (CHGUV) from March 2020 to April 2021. For the control group, eyes (n = 12) were previously collected from the General University Hospital of Alicante (HGUA) and the CHGUV. The Ethics Committee on Human Research of the General University Hospital Consortium of Valencia approved the study (84/2020). The procedures were carried out in compliance with the Declaration of Helsinki and informed consent for research was obtained from the donor’s family before procurance. Information about demographic data, sex, infection time, ocular and systemic pathologies, treatments during hospital admission, use of mechanical ventilation, laboratory biomarkers such as Sequential Organ Failure Assessment Score (SOFA), partial pressure of arterial blood oxygen/fraction of inspired oxygen ratio, PaO_2_/FiO_2_ (PaFI) and D-dimer values as well as clinical complications were gathered.

The number of retinas used for each analysis depended on the sample condition after processing and the availability of controls (Additional file 1: Table S1). No statistically significant differences were found for age between the COVID-19 and control groups (*P* > 0.05; Additional file 1: Table S1).

### Ocular dissection and retinal immunohistochemistry

Enucleation occurred 1–3 h after death and immediately after that the eyes were fixed in 4% paraformaldehyde (PFA) for 2 h at room temperature (RT). Then, samples were washed in 0.1 M phosphate buffer (PB) and cryoprotected in a sucrose gradient (15%, 20% and 30%) at 4 °C. Once the cornea, iris and lens were removed, the posterior pole was dissected into nine pieces, always keeping the fovea and optic nerve on the temporary-central portion. These portions were used to obtain 14 µm transversal sections with the cryostat and for wholemount analysis. Immunohistochemistry in wholemount retinas and transversal sections was performed (Table 1, Additional file 1: Table S1). Retinal sections and wholemounts were washed three times in PB and the primary antibody diluted in PB with 0.1% or 1% Triton X-100 was incubated overnight at RT or for 4–5 nights at 4 °C, respectively. Primary antibodies used for immunostainings are described in Table 1. Then, samples were washed in PB and incubated with the secondary antibody (Table 1) for 1 h at RT in retinal sections or 2 nights at 4 °C for wholemounts. Next, retinal sections were washed in PB and TO-PRO was added for 15 min at RT. The TO-PRO iodide was the commercial fluorochrome used for nuclei staining. Finally, preparations were washed again, mounted using Citifluor (Citifluor, London, UK) and coverslipped. Images were obtained with a Leica TCS SP8 confocal laser-scanning microscope (Leica Microsystems).

**Table 1:** Primary and secondary antibodies used in this study

Molecular marker	Antibody	Source	Working dilution
Calbindin D-28K	Rabbit polyclonal	Swant (CB-38a)	1:500
Recoverin	Rabbit polyclonal	Proteintech (10073-1-AP)	1:500
ACE2	Rabbit polyclonal	Abcam (ab15348)	1:200
CRALBP	Mouse polyclonal	Abcam (ab15051)	1:200
GFAP	Mouse polyclonal	Sigma (#G3893)	1:200
Iba-1	Rabbit polyclonal	WAKO (#019–19,741)	1:1000 retinal sections, 1:200 wholemounts retinas
Collagen type IV	Goat polyclonal	Millipore (AB769)	1:1000 retinal sections, 1:500 wholemounts retinas
AF555	Rabbit, mouse	ThermoFisher Scientific (A31572, A31570)	1:100
AF488	Rabbit, mouse	ThermoFisher Scientific (A210206, A21201)	1:100
AF633	Goat	ThermoFisher Scientific (A21082)	1:100
TO-PRO (NucRed Dead 647)	–	Invitrogen (R37113)	1:1

*ACE2 =* angiotensin-converting enzyme 2; *CRALBP =* cellular retinaldehyde–binding protein; *GFAP =* glial fibrillary acidic protein; *Iba-1 =* ionized calcium binding adaptor molecule 1; *AF =* Alexa Fluor

### Quantification of microglia cells

The total number of ionized calcium binding adaptor molecule 1 (Iba1)^+^ cells and the number of Iba1^+^ on the retinal vessels was counted in sectors of 1.5 × 1.5 mm in the temporal area of flat-mount retinas using confocal images (Additional file 1: Table S1). For the quantification, cells of the three different retinal vascular plexuses were considered.

The area occupied by the Iba1^+^ cells was assessed using ImageJ and confocal images of retinal sections. Two complete retinal sections (mosaic images) were imaged from each sample using the pinhole at 380 µm. The pinhole was wider to increase the focus depth and easily obtain most of the processes of microglial cells. The quantification was presented relative to the total area of the retinal section.

### Quantification of axon terminal area and axon width in cone photoreceptors

The terminal area of the cone photoreceptor was measured in two or more confocal images of 390 × 390 µm in the transversal sections using anti-calbindin antibody and a macro in ImageJ specifically designed for this purpose (Additional file 1: Table S1). Specifically, a width of 35 µm from the end of the cone pedicle to the axon was selected on the images for area quantification. Only the well-oriented retinal sections and with Henle fiber layer (HFL) were included in the analysis. The result was presented as the proportion of the cone terminal area in the retina relative to the total number of cones.

The width of the cone axon was quantified with Adobe Photoshop (version 22.5.1) at the midpoint of the axon in both groups (Additional file 1: Table S1). The cone photoreceptor had to appear almost complete on the confocal images and HFL had to be present before measurement of axonal width. Two or more images were used for the quantification and four or more cones in each image were measured.

### Cell death detection

In situ cell death detection kit (Roche) based on labeling of DNA strand breaks [(TdT-mediated dUTP-X nick end labeling (TUNEL)] was used to quantify apoptotic retinal cells (Table 1, Additional file 1: Table S1). Briefly, the DNA cleavage can be detected by labeling the free 3′-OH termini with fluorescein modified nucleotides in an enzymatic reaction. According to the manufacturer’s protocol, retinal cell death detection was conducted using 14 µm retinal cryosections from 4% PFA (w/v) fixed eyes (methods described before). After three washes with PB, slides were incubated in phosphate buffered saline (PBS) with 1% Triton X-100 (v/v) for 5–10 min at RT in humidity chamber. Then, TUNEL mix reagent was incubated for 1 h at 37 °C in dark conditions. Thereafter, the reaction was stopped with three PB washes for 5 min at RT in the dark. Finally, preparations were mounted using Citifluor and coverslipped.

### Patient severity score

Each patient in the COVID-19 group obtained a total severity score considering D-dimer, the PaFI(PaO_2_/FiO_2_) and the presence of other organ failure (SOFA values) (Table 2). This severity score was based on previous studies [16–18]. According to the total score, patients were classified in different stages of clinical severity: minor (0), moderate (1), or severe (2) disease. Several biomarkers were also analyzed (Additional file 1: Tables S2 and S3).

**Table 2:** COVID-19 disease severity scale

Scores	Parameters		
	PaFI (PaO_2_/FiO_2_)	SOFA	DD (ng/mL)
+ 0 point	> 400	≤ 3	–
+ 1 point	300–400	–	–
+ 2 points	200–299	3–5	> 10,000
+ 3 points	< 200	> 5	–

*PaFI =* partial pressure of arterial blood oxygen/fraction of inspired oxygen ratio; *PaO*_*2*_ = partial pressure of arterial blood oxygen; *FiO*_*2*_ = fraction of inspired oxygen; *SOFA* = sequential organ failure assessment; *DD* = D-dimer

No point was added if patients had a PaFI > 400 or SOFA ≤ 3. One point was added if they presented a PaFI between 300 and 400. If the PaFI was between 200 and 299, SOFA values were between 3 and 5 or D-dimer was > 10,000, 2 points were assigned for each. The absolute value of severity for each patient was obtained by adding the score for each parameter present. The score range was between 0 and 7 points: mild disease included patients with a score between 0 and 3, moderate disease was considered with a score of 4 points, and severe disease included patients with a score superior to 4 points

### Classification of retinal changes in cones, Müller cells and microglia: score system

Confocal images of retinal sections were acquired for calbindin D-28K, cellular retinaldehyde-binding protein (CRALBP), Iba1 and collagen type I antibodies to study the cone photoreceptor, Müller cells and microglia. Three or four sections of the inferior and temporal area close to the macula were assessed, and images of three or more areas of 390 × 390 µm were obtained throughout the section.

The classification of retinal cells was based on previous reports [19–21] and the immunohistochemical study we performed. The morphological observations were independently assigned by two retinal specialists (three in the case of microglial cells). For the determination of the cellular changes, all the cells in the images were considered excluding those areas where gaps in the tissue existed.

With respect to the cones, the score was defined considering the uniformity of the inner and outer segments, the axon width, and the pedicles. Müller cells were scored according to the honeycomb-like pattern and the external limiting membrane (ELM). The classification of microglia was established depending on the processes, the cell body, the location respect to the vessels and the area occupied by the microglia in the retina. Changes in these areas were used to define three different stages for each cell. Each stage implied a numerical mark (+ 0, + 1 or + 2). The total retinal degeneration score for each patient was obtained by adding the individual scores for each cell type.

### Statistical analysis

Statistical analysis was performed using IBM SPSS Statistics (version 27.0. Armonk, NY: IBM Corp) and GraphPad Prism (version 6.0.0, GraphPad Software, San Diego, California USA). The assessment of normality was carried out by Kolmogorov-Smirnov or Shapiro-Wilk tests. Statistical analysis was performed with the non-parametric Mann-Whitney U test to compare the COVID-19 with control group. To measure the statistical relationship among the retinal state with different demographic and clinical parameters in the COVID-19 group, Spearman's correlation coefficient and line regression were used. A *P* value of less than 0.05 was considered significant.

## Results

### Clinical findings

Sixteen human donor eyes from patients deceased by COVID-19 (80 ± 10 years; 43.75% women, *P* > 0.05) and 15 donor eyes (68 ± 8 years; 33.33% women, *P* > 0.05) were included. Due to the advanced age, 87.5% of patients affected by COVID-19 presented comorbidities (14/16) and the 31.3% (5/16) had some retinal pathology (Table 3). COVID-19 disease lasted an average of 20 ± 10 days and 43.8% (7/16) suffered complications (Table 3, Additional file 1: Table S3). When compared to the reference ranges for healthy individuals of IL6 (4.631–5.740 pg/mL) [22], the expression levels of this biomarker were increased for all SARS-CoV-2 patients even reaching a concentration of 679 pg/mL in one of the cases (Additional file 1: Table S3). In addition, most COVID-19 patients suffered from lymphopenia (14/16), and all of them show increased levels of C reactive protein and lactate dehydrogenase, which are established markers for the pathology [23–25] (Additional file 1: Table S3). For severity score in COVID-19 patients, 37.5% (6/16) of the patients had the lowest degree of severity, 25% (4/16) showed a moderate severity and 37.5% (6/16) reached the highest degree of severity (Table 3).

**Table 3:** Clinical description of study patients

ID patient	Age (years)	Sex	Ocular pathologies	Systemic comorbidities	Duration of illness (days from onset)	Mechanical ventilation	Clinical severity score*
CoV-1	84	M	None	Alcoholism	4	No	1
CoV-2	84	F	None	Alzheimer’s, kidney disease, arterial hypertension	2	Yes	–
CoV-3	83	M	Drusen	Hypertensive heart disease	19	No	0
CoV-4	64	M	None	None	32	Yes	2
CoV-5	71	F	None	None	36	No	0
CoV-6	67	M	None	Metastatic prostate cancer in radiotherapy treatment and androgen suppression therapy, arterial hypertension	16	Yes	2
CoV-7	63	M	POAG	Arterial hypertension, ulcerative colitis	45	Yes	2
CoV-8	90	M	None	Arterial hypertension, diabetes mellitus	15	No	2
CoV-9	90	M	None	Dyslipidemia, high blood pressure, benign prostatic hyperplasia, colonic neoplasia	12	No	2
CoV-10	88	M	Intermediate AMD	Alzheimer’s disease	23	No	0
CoV-11	91	F	Dry eye	Arterial hypertension, dyslipidemia, ischemic cardiomyopathy, Alzheimer’s disease, ictus	36	No	1
CoV-12	92	F	Geographic atrophy AMD	Arterial hypertension, dyslipidemia, COPD, benign prostatic hyperplasia	13	No	1
CoV-13	88	F	None	Dyslipidemia, Alzheimer’s disease, atrial fibrillation, ictus	4	No	–
CoV-14	98	F	None	Arterial hypertension, dyslipidemia, tachycardia arrhythmia,	16	No	0
CoV-15	84	M	Geographic atrophy AMD, POAG	Arterial hypertension, diabetes mellitus	40	No	2
CoV-16	91	F	None	Arterial hypertension, Alzheimer’s disease, atrial fibrillation	-	No	0
Control-1	72	F	None	Arterial hypertension	–	N/A	–
Control-2	70	M	None	Arterial hypertension, cardiopathy	–	N/A	–
Control-3	74	M	None	Arterial hypertension, auricular fibrillation, hyperuricemia	–	N/A	–
Control-4	64	M	None	None	–	N/A	–
Control-5	74	M	None	Arterial hypertension	–	N/A	–
Control-6	79	M	None	Arterial hypertension, myocardiopathy, lobectomy by bronchogenic carcinoma	–	N/A	–
Control-7	57	M	None	None	–	N/A	–
Control-8	72	F	None	None	–	N/A	–
Control-9	78	M	None	Arterial hypertension, benign prostatic hyperplasia, lung cancer	–	N/A	–
Control-10	77	M	None	N/A	–	N/A	–
Control-11	70	F	None	Oncologic patient	–	N/A	–
Control-12	53	F	None	N/A	–	N/A	–
Control-13	57	F	None	Arterial hypertension	–	N/A	–
Control-14	65	M	None	None	–	N/A	–
Control-15	62	M	None	None	–	N/A	–

*M* = male; *F* = female; *POAG* = primary open-angle glaucoma; *AMD* = age-related macular degeneration; *COPD* = chronic obstructive pulmonary disease; *N/A* = not available

*The severity score was determined by SOFA-score, PaFi values, and D-dimer. Some clinical data from CoV-2 and CoV-13 were not available

**Fig. 1:**
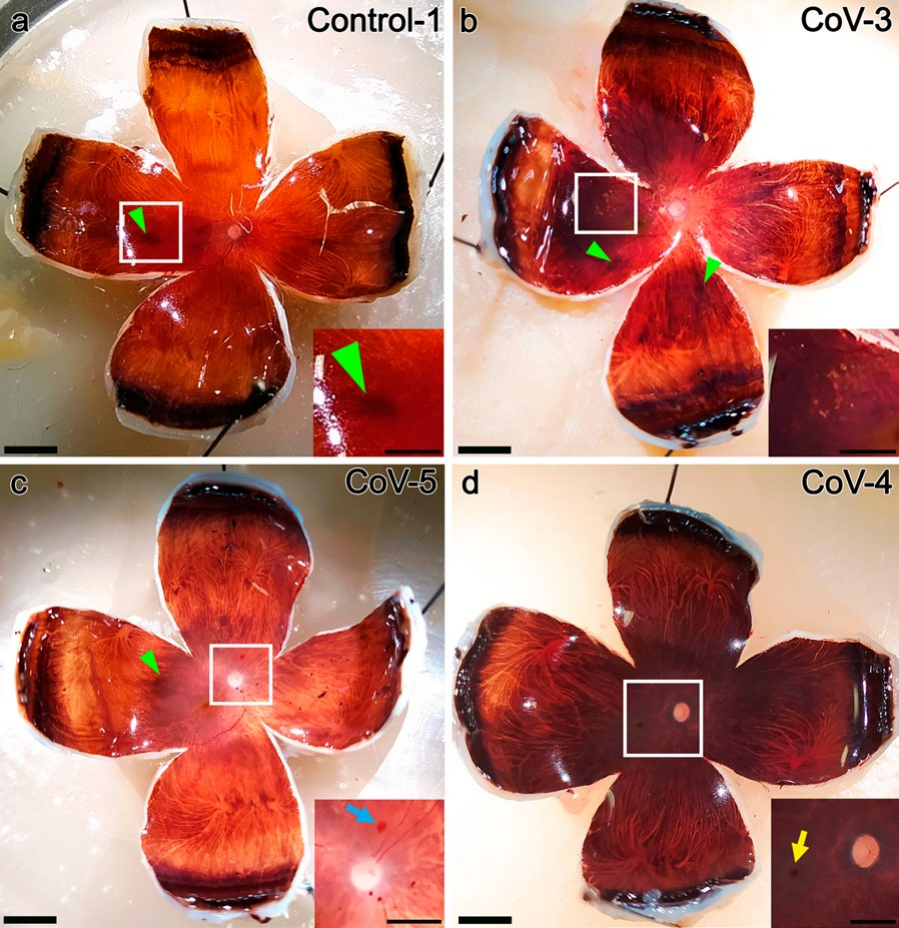
Representative eye fundus after dissection of ocular tissue in control (**a**) and COVID-19 patients (**b**–**d**). **a** Ocular tissue of a control patient with a slight hyperpigmented area in the temporal portion (inset, green arrowhead). **b**–**c** Dissection of samples from COVID-19 patients showing drusen deposits in the limits of the macula (**b**, white square, inset), hyperpigmented areas (**b**, **c**, green arrowheads) and a small flame-shaped hemorrhage superior to the optic nerve (**c**, blue arrow, inset). Mild peripapillary atrophy (**b**) and ischemia (**c**) can be also observed. **d** Eye fundus of a COVID-19 patient without remarkable alterations. Fovea (yellow arrow, inset) and optic nerve at higher magnification (inset). The choroid vessels (seen as white lines throughout the fundus) branch from the periphery to the fovea in all of them. Scale bar: 500 µm (insets: 250 µm)

The examination of the fundus eye (Fig. 1) revealed small hyperpigmented areas on the retinal pigment epithelium (RPE) (Fig. 1a–c, green arrowheads) in 16.6% of control eyes (1/6) and 43.75% of COVID-19 patients (7/16). Only one eye showed an isolated flame or dot-shaped hemorrhages close to the macula (Fig. 1c, blue arrow), 31.6% had peripapillary or peripheral atrophy (5/16), 12.5% showed parafoveal drusen (2/16) and one presented with a macular scar. Most of them lacked vascular or retinal alterations in the fundus at this view (Fig. 1d) with vascular tortuosity (12.5%) or copper wiring being two of the few signs observed. The only signs found in the control group were small areas of peripheral atrophy (1/6) and hyperpigmented areas on the RPE (1/6).

### Mild variations in the immunostaining pattern of ACE2 and disruption of Müller cells were found in COVID-19 patients

**Fig. 2:**
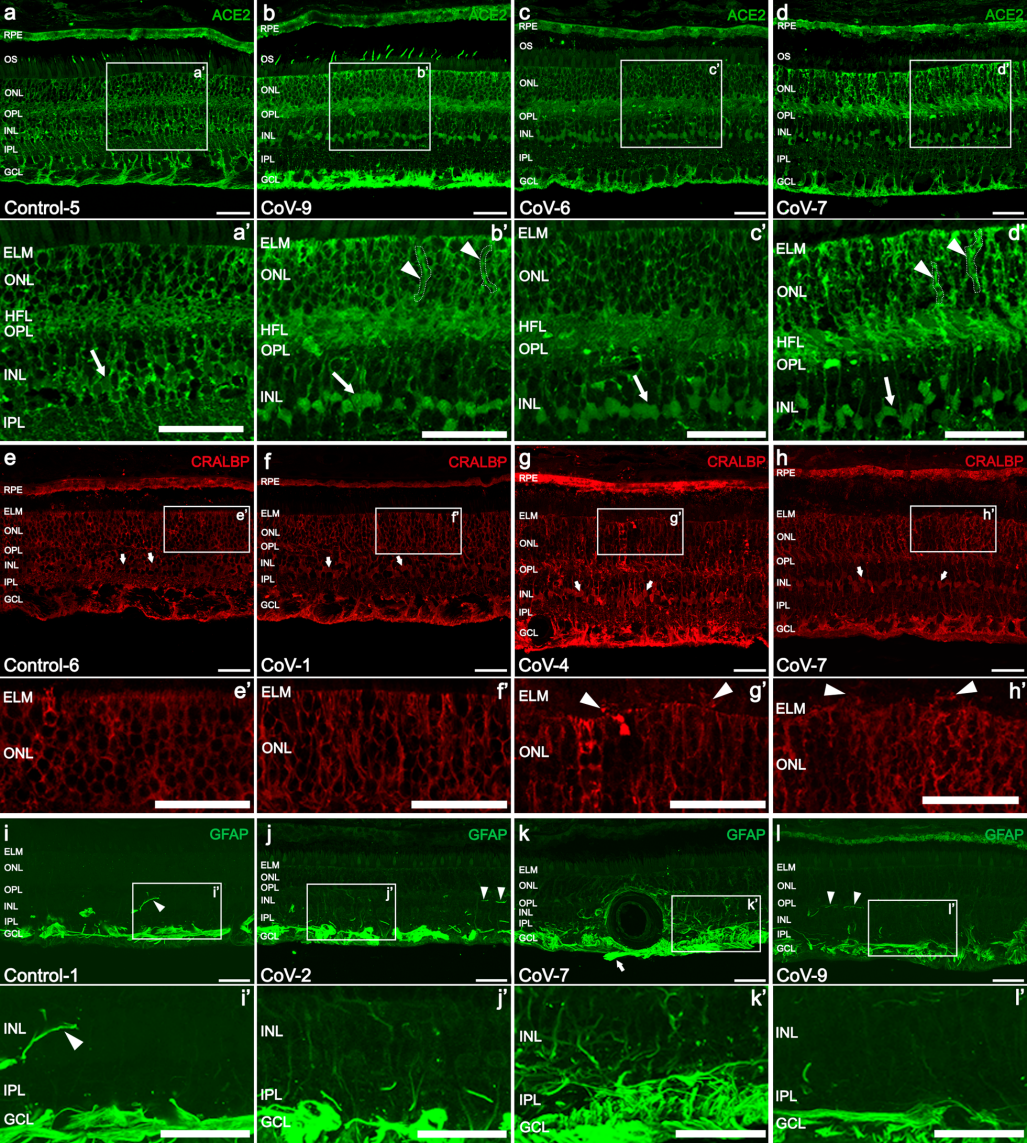
Vertical section of the retinas from control and COVID-19 patients immunolabeled with ACE2 (**a**–**d**), CRALBP (**e**–**h**) and GFAP (**i**–**l**). High magnification of selected area (white squares) allows detection of structural changes throughout the retina in COVID-19 patients compared with the controls (**a’, e’, i’** vs*.* **b’–d**’, **f’–h’**, **j’–l’**). **a–d** ACE2 protein was mainly present in Müller cells, OS of cones and RPE in both control and COVID-19 retinas. Lack of staining in OS of cones and greater staining at the level of ONL (arrowheads and white dotted areas) and cell body (arrows) was found in some COVID-19 retinas (**b–b’**, **c–c’**, **d–d’**) compared to the controls (a-a’). **e–h, e’–h’** Müller cells and RPE stained with antibody against CRALBP in COVID-19 patients showed signs of a disruption of the ELM (arrowheads), disorganization of honeycomb-like pattern and an increase of immunoreactivity in the cell body (arrows). **i–l** An increase in the staining of astrocytes appears to exist in COVID-19 retinas although differences in the morphology of astrocytes were not clearly observed. Astrocyte protrusion through the ILM was found (**k**, arrow). Increased reactive gliosis was observed in COVID-19 retinas (**j’**–**l’**) compared to the controls (**i’**). Arrowheads show astrocytes in the intermediate and deep capillary plexuses (**i**, **j**, **l**). ACE2, angiotensin-converting enzyme 2; CRALBP, cellular retinaldehyde-binding protein; OS, photoreceptors outer segments; RPE, retinal pigment epithelium; ELM, external limiting membrane; ONL, outer nuclear layer; HFL, Henle fiber layer; OPL, outer plexiform layer; INL, inner nuclear layer; IPL, inner plexiform layer; GCL, ganglion cell layer; ILM, internal limiting membrane; GFAP, glial fibrillary acidic protein; Scale bars: 50 μm

The ACE2 protein was mainly located in the Müller cells, RPE and in most outer segments of cone photoreceptors in both control and COVID-19 subjects (Fig. 2a–d). Only four patients infected with SARS-CoV-2 (25%) showed less immunostaining of ACE2 in the outer segment of photoreceptors than controls (Fig. 2c, d). ACE2 was barely found in the endothelium of retinal vessels. Disorganization of Müller cells structure at the level of outer nuclear layer (ONL) caused columns of increased ACE2 staining in 31.25% of COVID-19 patients (5/16) (Fig. 2a–d, a’–d’, some indicated with white dotted areas and arrows). In addition, the soma of Müller cells presented greater staining than controls (Fig. 2a–d, a’–d’, arrows). Since RPE cells were completely labeled by ACE2, differences were only observed when the epithelium was degenerated (Fig. 2d).

**Fig. 3:**
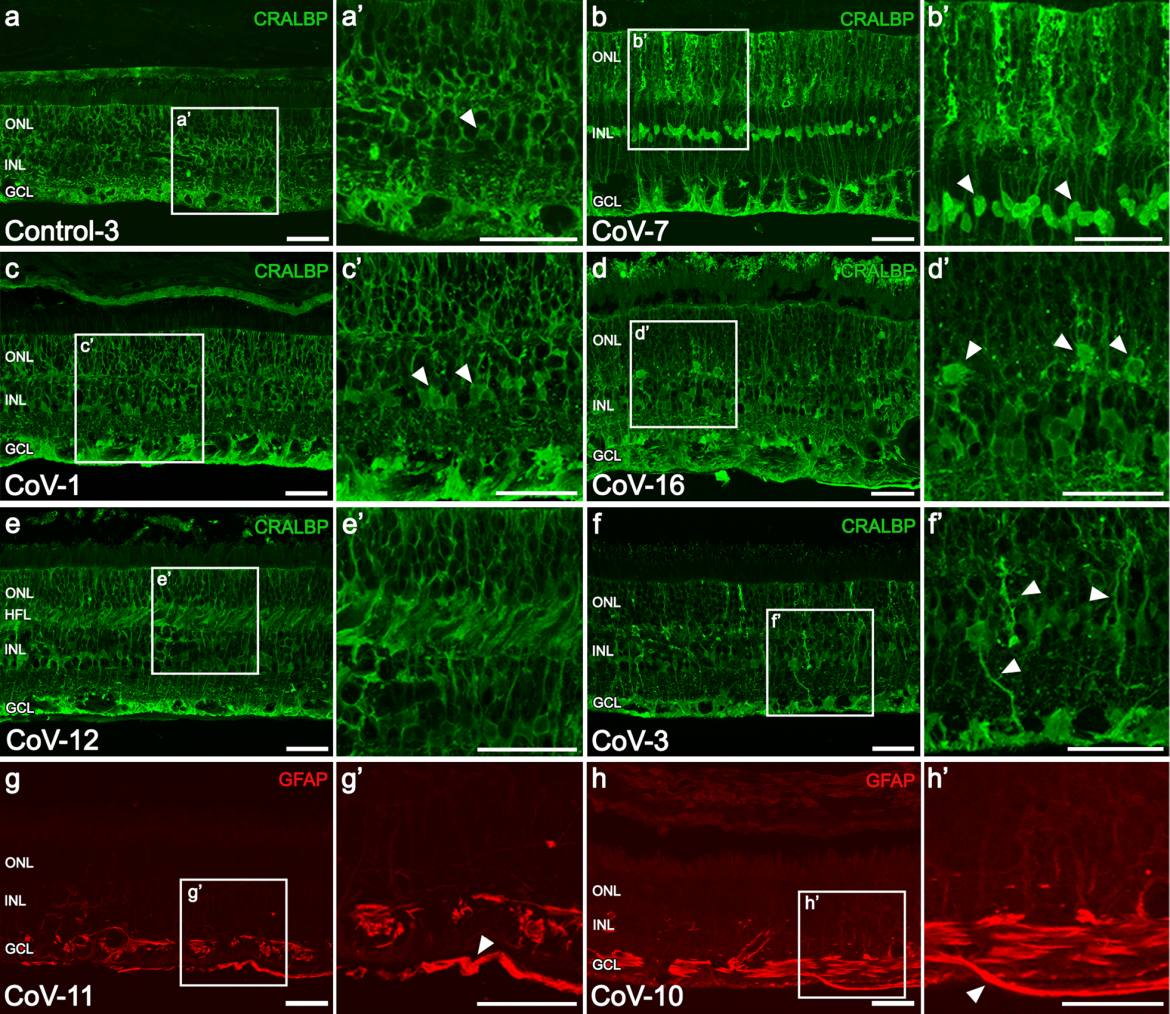
Morphological changes in Müller cells and astrocytes associated to glial activation. **a, a’** Normal morphology of Müller cells in a control subject. **b, b’** Greater staining in the honeycomb-like pattern at the ONL and soma (arrowheads) probably associated with an inflammatory process. **c, c’** Müller cells of a COVID-19 patient with normal morphology except for greater staining in the cell body (arrowheads). **d, d’** Translocation of cell bodies of Müller cells from the INL to the ONL (arrowheads). **e, e’** Swelling of Müller cells at the level of the HFL. **f, f’** Mild thickening of the main process in several cells (arrowheads). **g, g’** Fully formed epiretinal membrane (ERM) above the ILM labelled with GFAP (arrowhead). **h, h’** Astrocyte protruding the ILM (arrowhead) and starting the ERM formation. ONL, outer nuclear layer; HFL, Henle fiber layer; INL, inner nuclear layer; GCL, ganglion cell layer; CRALBP, cellular retinaldehyde-binding protein; GFAP, glial fibrillary acidic protein; Scale bars: 50 µm

Müller cells of COVID-19 retinas showed disorganization of ONL honeycomb-like pattern leading to strongly stained columns (6/16) compared to controls (1/6) (Fig. 2g, h vs. e; 3a, b). The cell body of these cells in the COVID-19 group also presented greater staining compared to controls (12/16 vs. 2/6) (Fig. 2g, h vs. e, arrows; 3b, c vs. a, arrowheads). Disruption of the ELM causing the sprouting of Müller cells towards the inner/outer segment was found in both the COVID-19 (4/16) and control groups (3/6) (Fig. 2g, h, g’, h’, arrowheads). The main morphological changes of Müller cells were classified in different stages (Table 4, Additional file: Table S4). Müller cells in stage 1 were present in 62.5% of COVID-19 patients and in 33.3% of the control group. Normal morphology (stage 0) was found in the 18.8% of COVID-19 retinas and in 50% of controls (Additional file 1: Table S4). The glial fibrillary acidic protein (GFAP) staining revealed reactive gliosis in 56.3% of COVID-19 patients (9/16) in contrast to controls (40%, 2/6) (Fig. 2i–l). In COVID-19 patients, Müller cells seemed to be more reactive around the vessels (Fig. 2k). Translocation of soma of Müller cells to outer nuclear layer (CoV-16, Fig. 3d–d’, arrowheads) and hypertrophied Müller cell processes at the level of HFL (31.25%, 5/16; Fig. 3e–e’) were also seen in the COVID-19 group. No translocation of the soma was found in the control group and 16.6% showed hypertrophied processes at the HFL level. The hypertrophy of Müller main process existed in both groups, being more frequent in the COVID-19 group (COVID-19: 66.67% vs. control: 40%; Fig. 3f–f’, arrowheads).

**Table 4:** Stages of response to damage in different cell types

Cell type	Stage 0—Normal morphology		Stage 1—Slight or moderate response		Stage 2—Severe response	
Cones		• Straight OS • Uniform width between the IS and the soma • Axon width is half the width of the soma/IS • Small flat pedicles		• Mild thinning of axon • Axon at HFL level is swollen		• Shorter OS • Voids in IS/OS or disorganization • Axon thinned at HFL level or/and pedicles swollen
Müller cells		• Honeycomb-like pattern defined • Stable ELM		• Honeycomb-like pattern is perceived but has disorganized areas • Isolated alterations of the ELM • Columns with greater staining		• Honeycomb-like pattern disorganized: structure of nuclei photoreceptor is not perceived • Frequent disruptions of ELM • Areas with higher staining
Microglia		• Resting microglia • Long ramification • Area occupied by the microglia > 2% of the retinal section		• Various morphologies: resting microglia, ameboid-shape or hypertrophic cells are observed • Retraction of ramifications • Area occupied by the microglia 1.3%–2% of the retinal section		• Ameboid-shape, phagocytic or hypertrophied cells • Retracted branches • Mainly located in retinal vessels • Area occupied by the microglia < 1.3% of the retinal section

*OS* = outer segment; *IS* = inner segment; *HFL* = Henle fiber layer; *ELM* = external limiting membrane

Finally, morphological characteristics of astrocytes were preserved in both groups (Fig. 2i, l). Nevertheless, 50% of COVID-19 patients (8/16) presented astrocytes protruding to the vitreous humor from the ganglion cell layer or epiretinal membranes (ERMs) (Figs. 2k arrow; 3g–g’, h–h’, arrowhead). No ERM or astrocytes protruding were observed in the control group. The staining of the inner nuclear layer (INL) and the outer plexiform layer (OPL) corresponds with the presence of astrocytes located near the retinal blood vessels of the intermediate capillary plexus (Fig. 2i, j, l, arrowheads).

### Microglia activation was confirmed in retinas of COVID-19 patients

**Fig. 4:**
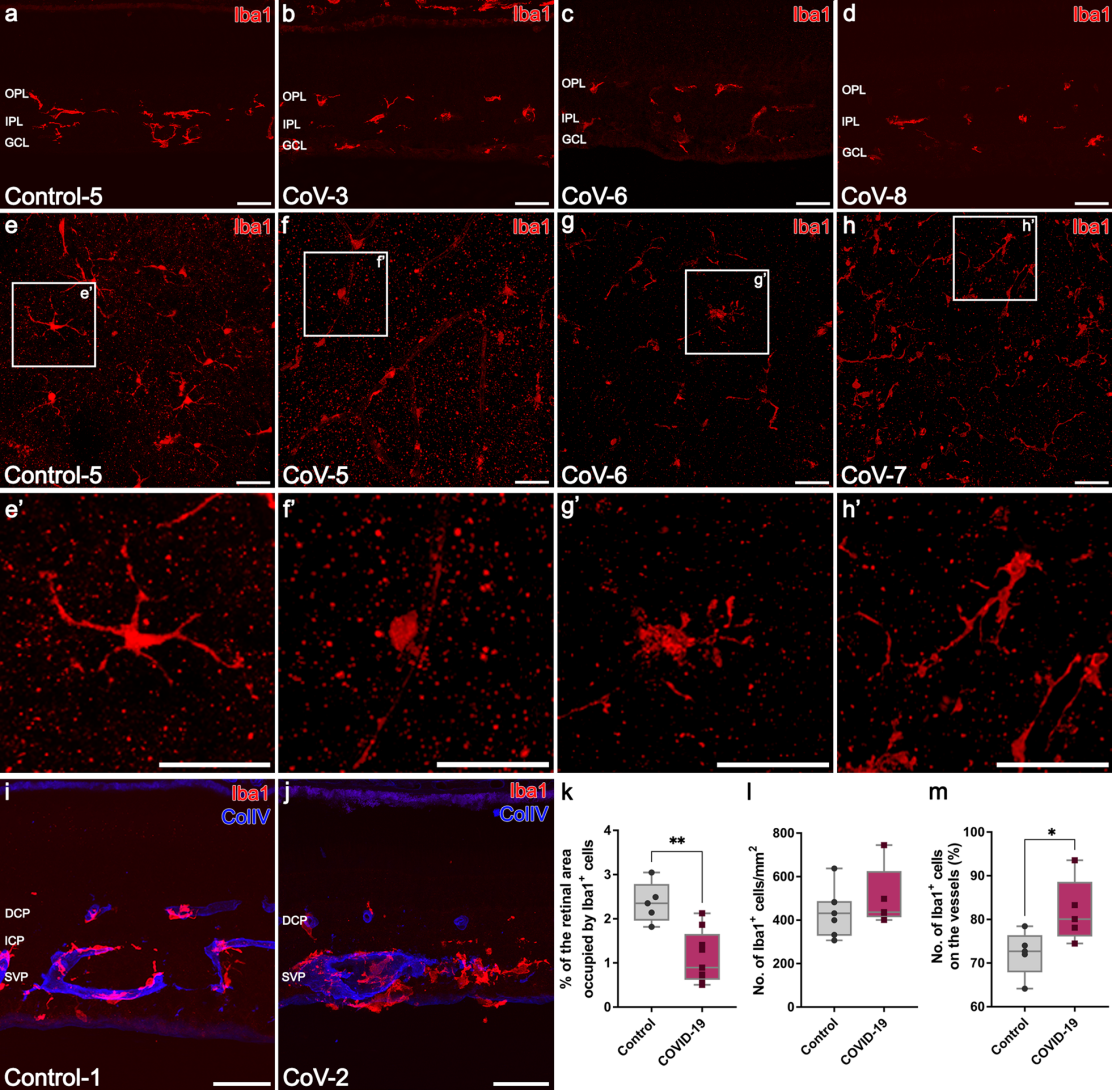
Immunolabeling with Iba1 on retinal sections (**a**–**d**, **i**–**j**) and flat-mount retinas (**e**–**h**) from control (**a**, **e**, **i**) and COVID-19 (**b**–**d**, **f**–**h**, **j**) patients reveals microglia activation. Note the morphological changes of microglia cells in the high magnification insets (**e’**–**h’**, white square) where the shape of cells in the OPL changed from ramified in controls (e’) to ameboid or with a medium retraction of its processes in COVID-19 (**f’**–**h’**) patients. **i, j** Differences in the microglia shape around the vessels in a control subject (**i**) and the clear nodules of microglia in a COVID-19 patient (**j**). **k** Proportion of microglial cells around the vessels in the COVID-19 (n = 5) and control (n = 5) patients (*P < 0.05). **l** Graphical representation of the area covered by Iba1-positive cells in retinal sections of COVID-19 patients (n = 9) compared with the control group (n = 5) (**P < 0.01). **m** There are no differences in the mean of Iba1-positive cells density quantified in the three retinal vascular plexuses between control (n = 7) and COVID-19 (n = 7) flat-mount retinas (*P* = 0.3434). OPL, outer plexiform layer; IPL, inner plexiform layer; GCL, ganglion cell layer; Iba1, ionized calcium binding adaptor molecule 1; Col IV, collagen type IV; Scale bars: 50 µm

**Fig. 5:**
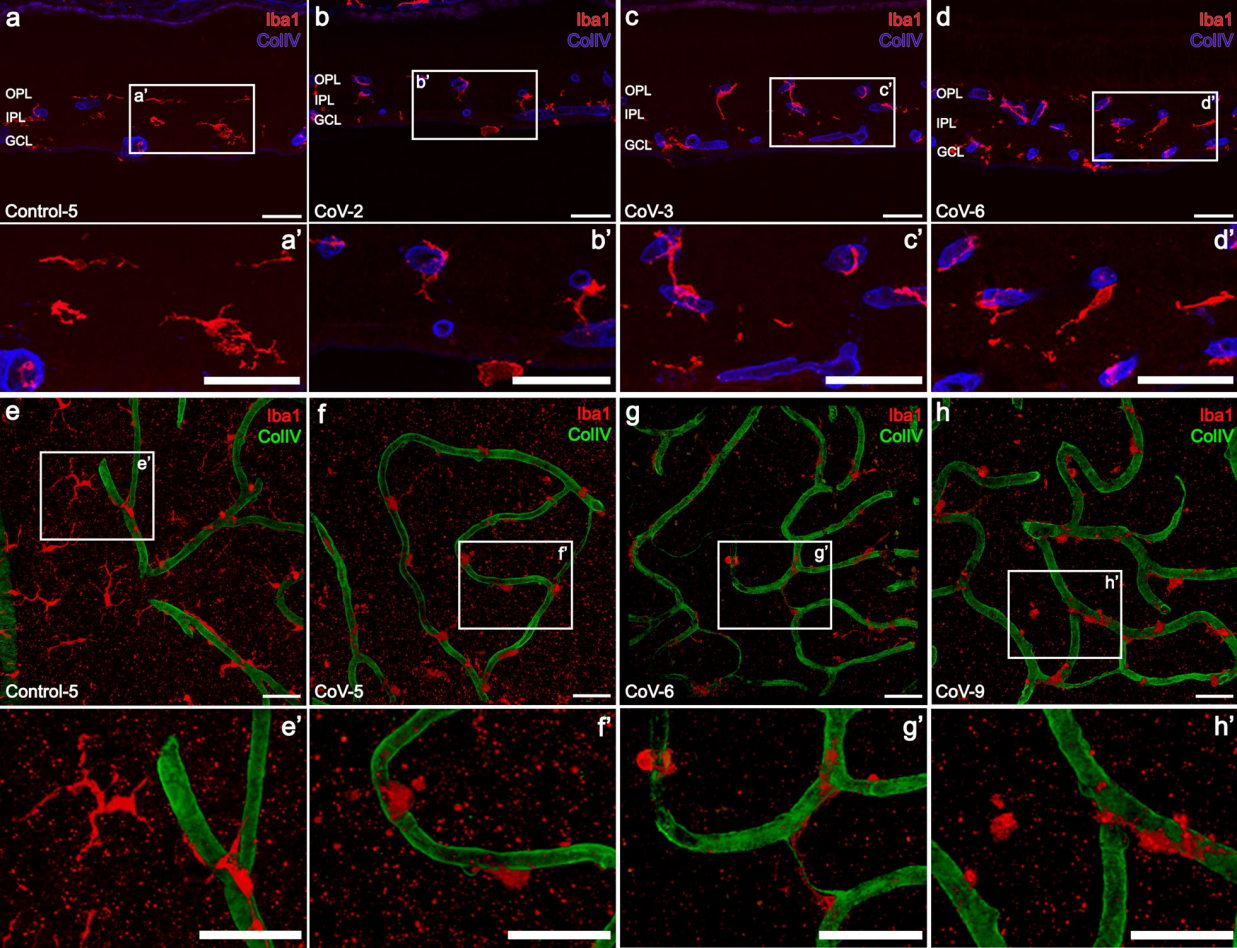
Representative images from control (**a**, **e**) and COVID-19 (**b**–**d**, **f**–**h**) retinal sections, immunolabeled with Iba1 (red) and collagen type IV (blue) to detect microglia migration towards the vessels. **a–d, a’–d’** Most of the microglia cells in COVID-19 patients were found surrounding the vessels compared to controls (**a** vs*.* **b**–**d**). **e–h** Flat-mount retinas at the outer plexiform layer showing greater migration of microglial cells toward retinal vessels in COVID-19 patients (**f**–**h**) compared to controls. **e’-h’** Note not only the migration process but also the morphological changes of the microglial cells due to their activation in COVID-19 patients compared to healthy one (**f’**–**h’** vs*.* **e’**). OPL, outer plexiform layer; IPL, inner plexiform layer; GCL, ganglion cell layer; Iba1, ionized calcium binding adaptor molecule 1; Col IV, collagen type IV; Scale bars: 50 µm

Microglia activation was confirmed in most of the retinas of COVID-19 patients studied, showing areas where a remarkable number of microglia changed from a ramified resident morphology (Fig. 4a, e) to an ameboid or activated shape (93.8%, 15/16) (Fig. 4b-d, f–h). In contrast, only 33.3% (2/6) of the control retinas presented regions with a high number of ameboid-shape microglia. Ameboid cells lack processes and showed a relatively small cell body (Fig. 4b, f–f’). Hypertrophied microglia and cells with a medium retraction of their processes were also found in COVID-19 patients (Fig. 4c–d, g–g’, h–h’). These activated microglia were also observed in the flat-mount retinas of COVID-19 patients, especially in the outer plexiform layer (Fig. 4f-h, f’–h’). Another characteristic sign found in these patients was the presence of nodules of microglia around large vessels (Fig. 4i, j). Changes in morphology were confirmed since the area occupied by all the microglial cells was significantly reduced in COVID-19 patients (*P* < 0.01; Fig. 4k). In fact, the area occupied by the microglia in 55.55% of the COVID-19 retinas was reduced by more than 50% with respect to the area occupied in controls. Also, 33.33% of the retinas from COVID patients showed a reduction between the 20–50% of the area occupied by microglia respect the retinas from control group. Significant differences in the total number of Iba1^+^ cells were not observed compared to controls (*P* = 0.3434; Fig. 4l). Moreover, an increase in the proportion of microglial cells around the vessels in COVID-19 patients in all vascular plexuses existed compared with control (*P* < 0.05; Fig. 4m; Fig. 5a, e–e’ vs. b–d, f–h). Three stages were also established with respect to microglia response to damage (Table 4; Additional file 1: Table S4).

### COVID-19 retinas showed swelling at the axon terminal of cone photoreceptors but cell death was not detectable

**Fig. 6:**
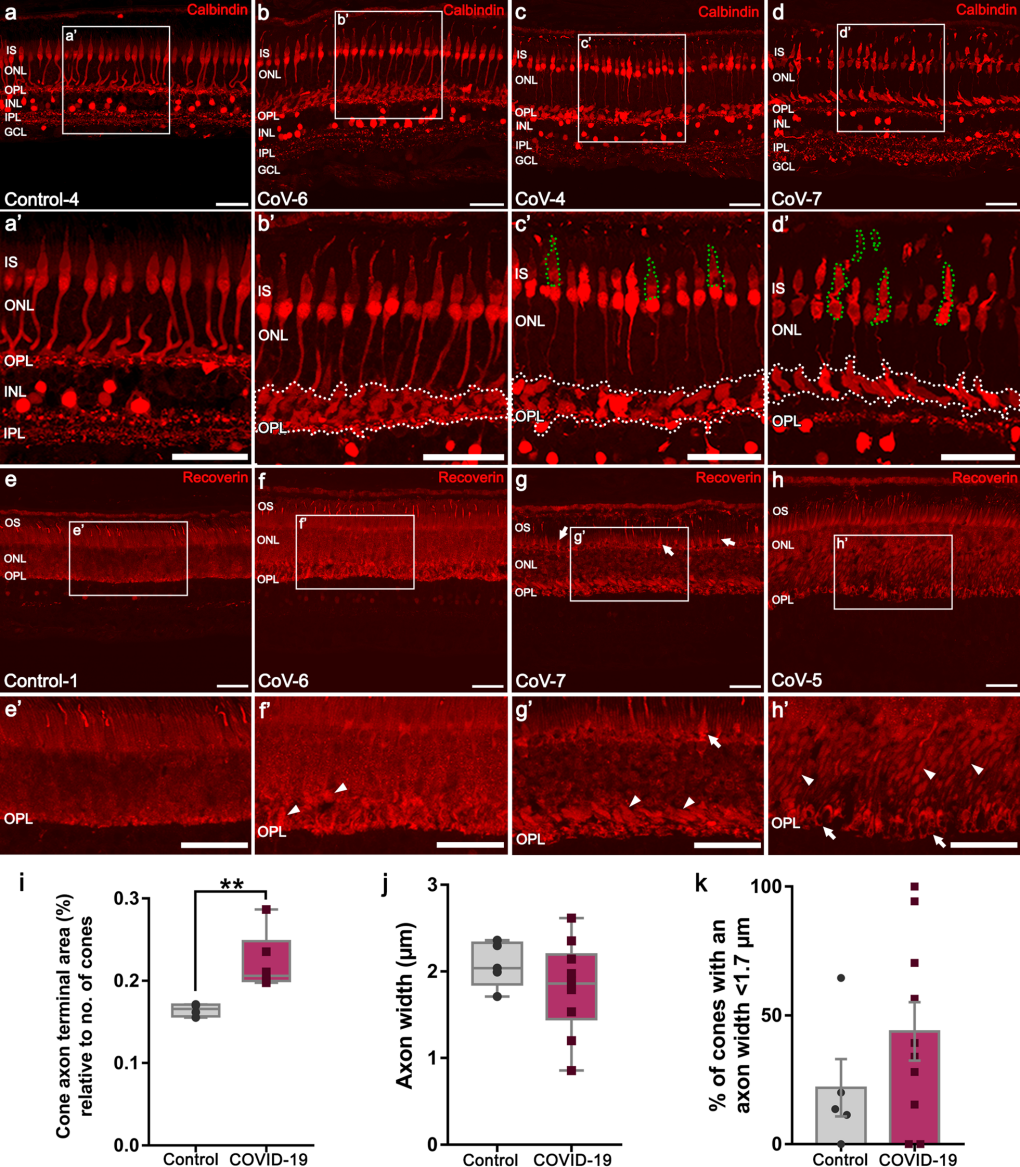
Retinal sections from control and COVID-19 patients immunostained with calbindin (**a**–**d**) and recoverin (**e**–**h**). **a, a’** Cones with a normal morphology are labeled. Outer and inner plexiform layer and some horizontal, cone bipolar, amacrine and ganglion cells are also marked with calbindin. **b, b’** Cones showing a moderate response to damage: slight thinning of the axon which appears swollen at the HFL level is observed in some cells (**b’** and white dotted area). **c, d** Severe degeneration of most cones with an excessively thin axon that swells at the HFL (**c’**, **d’** and white dotted areas). Several alterations in the inner and outer segment were present (**c’**, **d’**, some indicated with green dotted areas). Degeneration between the cell body and the inner segment of cones and absence of pedicles were also found in some cases (**d’**). **e, e’** Normal pattern of recoverin staining photoreceptors and OFF cone bipolar cells. (**f–h**) Less dense recoverin staining pattern in the ONL and mild swelling of the axon at the HFL (**f’**, **h’**, arrowheads) and ONL (**g’**, arrowheads) level were present in some retinas of COVID-19 patients. Alterations in the OS/IS (**g**–**g’**, green dotted areas) and in the axon terminals (**h**–**h’**, arrows) were also detected. **i** Proportion of the terminal area of cones in the retina relative to the number of cones between COVID-19 (n = 6) and control group (n = 4) (**P < 0.01). **j** Axon width values of the control (n = 5) and COVID-19 groups (n = 10) (*P* = 0.309). **k** Percentage of cones with an axon width inferior to 1.7 µm in control (n = 5) and COVID-19 (n = 10) patients (*P* = 0.252). OS, outer segment; IS, inner segment; ONL, outer nuclear layer; OPL, outer plexiform layer; INL, inner nuclear layer; IPL, inner plexiform layer; GCL, ganglion cell layer; HFL, Henle fiber layer. Scale bars: 50 µm

Cone photoreceptors of retinas of COVID-19 patients showed a common degeneration pattern (Fig. 6). A moderate swelling of the axon at the level of the HFL appears to be the first degenerative sign (Fig. 6b, b’, white dotted area): the proportion of the terminal area of cones in the retina relative to the number of cones in the COVID-19 group was 0.22 ± 0.03% compared to the area of 0.164 ± 0.007% in the control group (*P* < 0.01; Fig. 6i). No differences were observed in the axon width of the cones between COVID-19 (1.8 ± 0.5 µm) and control retinas (2.1 ± 0.3 µm; *P* = 0.309; Fig. 6j). However, in six COVID-19 patients (6/10), more than 30% of cones presented with an axon width inferior to 1.7 μm, whereas this result was only found in one control retina (1/5) (Fig. 6k). Some retinas showing this axonal thinning and swelling at the HFL are shown in Fig. 6c’–d’. Degeneration of the inner/outer segment characterized by a narrowing of the outer segment, and an atrophy of the inner segment was also present in six COVID-19 retinas (37.5%; 6/16) and in two control retinas (33%; 2/6) (Fig. 6c–d, some indicated with green dotted areas, and Fig. 6g). Axonal swelling (Fig. 6f–h, f’–h’, arrowheads) and degeneration at axon terminals (Fig. 6h–h’, some pointed with arrows) are also shown. These alterations were used to establish the different stages of response to damage (Table 4; Additional file 1: Table S4). Specifically, 37.5% of patients with the coronavirus had a severe degeneration (stage 2) of cone photoreceptors compared to the 16.7% of control group (Additional file 1: Table S4). Even though cone degeneration exists, cell death was not detected in any group with the use of TUNEL assays (Additional file 1: Fig. S1).

### Total retinal degeneration increased in COVID-19 patients

**Fig. 7:**
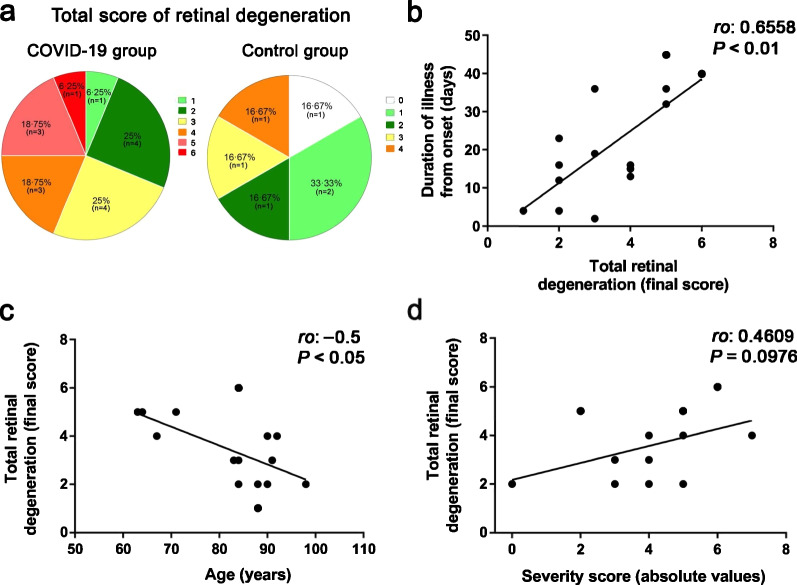
Total retinal degeneration and clinical correlations. **a** Graphics of total retinal degeneration in each group: the value of “0” means the absence of retinal alterations and the score of “6” corresponds to the most degenerated stage. The 43.75% of COVID-19 patients presented a retinal degeneration score between 4 and 6 (left) whereas only 16.67% of control group reached the highest score (four points, right). **b** Linear regression showing significant correlation between the duration of the disease and total retinal degeneration in the COVID-19 group. **c** Age correlates inversely with total retinal degeneration in the COVID-19 group. **d** Linear regression of the clinical severity with the total retinal degeneration. Spearman's correlation coefficient (*ro*) was performed in the COVID-19 group

Patients were grouped according to the total retinal degeneration score (Table 5). The total retinal degeneration score was obtained by adding the individual scores for each cell type studied; thus, the score of “0” meant the retina has no alterations and the score of “6” corresponded to the most degenerated retina (Fig. 7a, Table 5). The 25% (n = 4) of COVID-19 patients (CoV-4, 5, 7, and 15) had the highest retinal degeneration with 5 or 6 total points. However, no patient in the control group reached such a high score for retinal degeneration. Four points of retinal degeneration were found in 18.8% (n = 3) and 16.6% (n = 1) of the retinas from COVID-19 and control group respectively. Three of four patients with mechanical ventilation (CoV-4, 6 and 7) were the youngest in the group and reached a total retinal degeneration score between 4 and 6 along with the highest degree of severity. Only CoV-7 presented with a treated retinal disease. Two other patients with a severe health condition also showed a retinal degeneration score between 4 and 6 (CoV-8 and 15); these were older, but both had diabetes and one presented an end-stage retinal disease (atrophic age-related macular degeneration). The 66.67% of the retinas in the control group had retinal degeneration scores between 0 and 2 (n = 4) whereas only 31.25% (n = 5) of the retinas of COVID-19 group obtained a score between 1 and 2.

**Table 5:** Retinal findings and severity score in studied patients

ID patient	Age (years)	Sex	Retinal cell alterations score				Clinical severity score
			Cones	Müller cells	Microglia	Total degeneration	
CoV-1	84	M	0	0	2	2	1
CoV-2	84	F	0	1	2	–	–
CoV-3	83	M	0	1	2	3	0
CoV-4	64	M	2	2	1	5	2
CoV-5	71	F	2	1	2	5	0
CoV-6	67	M	1	1	2	4	2
CoV-7	63	M	2	2	1	5	2
CoV-8	90	M	1	1	2	4	2
CoV-9	90	M	0	0	2	2	2
CoV-10	88	M	0	1	1	2	0
CoV-11	91	F	1	1	1	3	1
CoV-12	92	F	2	1	1	4	1
CoV-13	88	F	0	0	1	–	–
CoV-14	98	F	0	1	1	2	0
CoV-15	84	M	2	2	2	6	2
CoV-16	91	F	2	1	0	3	0
Control-1	72	F	1	1	0	2	–
Control-2	70	M	1	2	1	4	–
Control-3	74	M	0	0	1	1	–
Control-4	64	M	0	0	0	0	–
Control-5	74	M	1	0	0	1	–
Control-6	79	M	2	1	0	3	–

According to Table 4, the score of the retinal cell types differentiates a normal morphology (0) from a mild-moderate (1) or severe (2) response to damage. Total retinal degeneration corresponds with the sum of the scores obtained in each cell type. The severity score corresponds with a mild (0), moderate (1) or severe (2) disease

*M* = male; *F* = female

A strong significant correlation among the Müller cells and cones alterations seems to exist; increased Müller cells activation leads to greater cone degeneration (*ro*: 0.680; *P* < 0.01). This correlation was not found neither between Müller cells and microglia activation (*ro*: − 0.186; *P* = 0.641) nor between cone photoreceptors and microglia activation (*ro*: − 0.258; *P* = 0.331, Additional file 1: Table S5 and Fig. S2).

A significant correlation was found between the total retinal degeneration and the duration of illness (*ro*: 0.6558; *P* < 0.01; Fig. 7b). The stage of response to damage of cone photoreceptors and Müller cells also correlated significantly with the duration of disease (*ro*: 0.647; *P* < 0.05 and *ro*: 0.7577; *P* < 0.01, respectively) (Additional file 1: Table S5 and Fig. S3). Moreover, total retinal degeneration correlated inversely and significantly with the age of patients of COVID-19 group (*ro*: − 0.5; *P* < 0.05). Graphical regression of these parameters (Fig. 7c) showed that most patients older than 88 years presented with a retinal degeneration score of 3 or less.

A tendency to correlation among total retinal degeneration and the severity of the disease (*ro*: 0.4609; *P* = 0.097) was observed (Fig. 7d). CoV-4 and CoV-7 presented a high retinal degeneration score (5 points) and the highest clinical severity. Similarly, with CoV-15’s retina had the most degeneration (6 points) but with a moderate severity. Interestingly, microglia score showed a moderate inverse correlation with PaFI values (*ro*: 0.6325; *P* < 0.05), showing that the lower respiratory capacity of the patient, the greater the activation of microglia cells (Additional file 1: Fig. S3). Finally, although no correlations were found between the microglia score and other parameters, in some cases, an increased activation of microglia coincided with the highest degree of clinical severity (CoV-6, 8, 9 and 15).

## Discussion

To date, COVID-19 related retinopathy has been mainly studied through non-invasive techniques such as retinography, optical coherence tomography (OCT) or OCT angiography (OCTA). The main findings detected in the fundus eye were cotton wool spots, micro-hemorrhages, and vessel tortuosity [15, 26]. Furthermore, a decrease in the foveal vascular density, an enlargement of the foveal avascular zone, and an increase in the diameter of the main veins and arteries were also observed [15, 26–28]. Despite these alterations, the only visual symptoms described are burning sensation or photophobia [15]. Herein, we graded the glial activation along with the morphological alterations of cone photoreceptors in COVID-19 retinas and correlated them with different clinical parameters.

In SARS-CoV-2 infected patients, the photoreceptors showed an axonal thinning with a more aggressive swelling of the axon at the HFL than previously described due to aging or other diseases [29]. Although we cannot completely disregard age-induced swelling in the axon terminals of the photoreceptors, in the case of our COVID-19 patients, the morphological changes we observed were more severe than what was previously described [29, 30]. Photoreceptors are the most susceptible cells to hypoxia and are closely associated with Müller cells. These glial cells regulate homeostasis and retinal blood flow [31–33]; thus, alterations in Müller cells might have an effect in photoreceptor degeneration as shown by strong correlation.

In COVID-19 retinas, Müller cells showed hypertrophic processes similar to what is observed with glial remodeling in the human retina [19] but in these patients, this sign was localized to the HFL level. This is probably due to the disruption of water transport since this retinal region is susceptible to these alterations [34]. Although retinal extracellular edema was not found in our samples [27] we observed signs that can precede the macular edema such as the ERMs [35]. This membrane could mean the beginning of an inflammatory process in the retina developed by a temporary vascular inflammation or an increase in cytokines [35, 36]. Finally, the astrocytes protruding into the vitreous humor to form the ERM have been also described in other retinal pathologies [37].

Gliosis of Müller cells observed with GFAP in the retina or around the large vessels may be due to the increased vascular permeability associated to hypoxic conditions of COVID-19. The upregulation of GFAP in these cells may act as a protective response [38].

The increased staining of the ACE2 protein may be another sign of Müller cells activation and could be related to the upregulation of the protective ACE2/Ang1-7/Mas axis of the renin-angiotensin system [39]. This protein has been previously detected in some layers of the human inner retina [40], but we demonstrate for the first time its predominant location in human Müller cells. On the other hand, the abundance of circulating cytokines in the bloodstream like IL6 (Additional file 1: Table S3) associated to the systemic inflammation could cause the degeneration of the endothelial cells with a reduction in the ACE2 protein, promoting the breakdown of the blood-retinal barrier (BRB). In fact, we barely found endothelial cells stained with ACE2, which could support the presence of endothelial damage. However, regarding the relationship among the endothelial cells and the ACE2, previous studies showed that the spike protein of SARS-CoV-2 over the endothelial cells may cause the up or downregulation of ACE2 [41, 42].

Reactive changes in the microglia of the retina have been described in other viral infections in animal models [43, 44]. In our study, microglia activation was prominent in COVID-19 retinas compared with age-matched controls, which is in accordance with Jidigam and colleagues’ study [27]. However, while they described them as hypertrophic or dystrophic cells, our results showed that microglial cells suffer different activation stages (ameboid-shape cells with a small cell body or microglia with a medium retraction of their processes). Moreover, we were not able to find an increase in the number of microglial cells either. Taken together, these results indicate that the cellular response to damage induced by COVID-19 varies among patients. We discarded that the microglia activation observed in our study was due to aging since the age-related changes found in the brain described dystrophic microglia [20, 45]. Furthermore, in the primate retina, there were no morphological changes in the microglia associated with aging [46], although these findings remain to be studied in detail for the human retina. We found microglial nodules around the retinal vessels of the superficial vascular plexus like those found in the brains of COVID-19 patients [6, 8] and an increase in the proportion of these cells around the vessels in all vascular plexuses. The increase of pro-inflammatory factors in the bloodstream of these patients along with the breakdown of the BRB may allow the access to the retina, promoting the activation and migration of the microglial cells [31].

Microglia could be activated increasing their response if previous retinal diseases exist [47, 48]. A previous inflammatory state in which microglial cells are already active [47, 48] may have caused the most ameboid-shaped cells in CoV-3, 8 and 15, patients with diabetes, drusen and age-related macular degeneration, respectively. In addition, the rest of the COVID-19 patients with a severe activation of microglia (CoV-1, 2, 6 and 9) also suffered different diseases such as alcoholism, Alzheimer’s disease, cancer, or neoplasia. A greater cone degeneration was observed in the CoV-7, 12 and 15, patients diagnosed with glaucoma or geographic atrophy.

Thakur et al. proposed that the microglial activation in the brain is more probably related to the hypoxia or the systemic response against the infection than the virus itself [8]. Therefore, these processes probably also occur in the retina since patients with lower oxygen exchange (lower PaFI values) presented with greater microglia activation. Hypoxia can also affect retinal cells [31]: a severe degeneration of cone photoreceptors may have occurred in CoV-4 and 5 due to low PaFI levels since they did not have any previous pathology. Generally, retinal degeneration was more severe with longer periods of infection with COVID-19.

Finally, age seems to be one of the most significant risk factors for developing severe forms of COVID-19 symptoms [49–51]. Despite that, our results showed that older patients presented with lower retinal disturbances. This may be related to the immunosenescence and the fact that age causes a poor immunological response against the virus [49, 50], causing the microglia in the retina to become activated to a lesser degree when compared with younger subjects. Therefore, the fact that retinal degeneration was inversely correlated with the age of patients makes it plausible that the retinal alterations observed in COVID-19 patients are due to the disease itself. On the other hand, patients in their 60s to 70s may develop an uncontrolled response bringing about an exaggerated inflammation in the tissues [51]. Three of five patients who showed a severe health condition and a high retinal degeneration score (between 4 and 6) were the youngest and were on mechanical ventilation. However, it is unknown if all these retinal alterations can affect the retinal function since apoptosis was not detected in any of our studied retina and no visual alterations have been described in patients with COVID-19 [15].

Several strengths can be found in this study. It is the largest study describing morphological changes in different retinal cells, including the specific location of ACE2 in the human retina. Additionally, we classified the cellular alterations with different degrees to evaluate more precisely the results and established correlations among retinal degeneration with clinical parameters. However, this study is not exempt of limitations. Due to the difficulty in getting human eye donors during the pandemic in Spain, all samples collected had to be included in this work (ocular disease was not an exclusion criteria). Despite having age-matched COVID-19 and control patients, the lack of laboratory data from control patients did not allow us to establish a comparison between groups.

## Conclusions

Retinal alterations of COVID-19 patients studied included microglia activation with an ameboid-shape and Müller cells gliosis along with changes in cone morphology. Different stages of the cellular response to damage were established considering these retinal changes. The correlation between clinical severity and the retinal degeneration score showed that the duration of the disease, age and PaFI affected the level of retinal impairment. Hence, all these retinal changes may be due to the systemic inflammation and hypoxia seeing that other reports did not describe changes in visual function. Additionally, COVID-19 could aggravate the retinal degeneration in patients with previous retinal diseases.

## Supplementary Information

**Additional file 1: Table S1.** Number of samples used for each analysis. **Table S2.** Laboratory data of COVID-19 patients and score defined to each value. **Table S3.** Biomarkers, complications and treatments during hospital admission of COVID-19 patients. **Table S4.** Frequency of observations in the immunohistochemistry images. Table S5. Retinal and clinical data used in Spearman's correlation coefficient. **Fig S1.** Detection of cell death by TUNEL assay. **Fig S2.** Linear regression and Spearman's correlation coefficient (ro) of the different retinal cells. **Fig S3.** Linear regression and Spearman's correlation coefficient (ro) between the retinal cells and clinical data.

## Data Availability

All relevant data are in the manuscript together with its supporting files.

## Abbreviations

ACE2: Angiotensin-converting enzyme 2
BRB: Blood-retinal barrier
COVID-19: Coronavirus disease 2019
ELM: External limiting membrane
ERM: Epiretinal membrane
GFAP: Glial fibrillary acidic protein
HFL: Henle fiber layer
INL: Inner nuclear layer
ILM: Internal limiting membrane
OCT: Optical coherence tomography
OCTA: Optical coherence tomography angiography
ONL: Outer nuclear layer
PaFI: Partial pressure of arterial blood oxygen/fraction of inspired oxygen ratio, PaO2/FiO2
POAG: Primary open-angle glaucoma
RPE: Retinal pigment epithelium
SARS-CoV-2: Severe acute respiratory syndrome coronavirus 2
SOFA: Sequential Organ Failure Assessment Score
TUNEL: TdT mediated dUTP-X nick end labeling
WHO: World Health Organization
