# Supplementary material for: Microglia activation and neuronal alterations in retinas from COVID-19 patients: correlation with clinical parameters
Source: Eye Vis (Lond). 2023 Mar 1;10:12. doi: 10.1186/s40662-023-00329-2 (PMC9974399; doi:10.1186/s40662-023-00329-2)

**Supplementary information**

**Table S1. Number of samples used for each analysis.**

|  | **Number of samples used** (**mean age of donors, years** (**y**)) | | |
| --- | --- | --- | --- |
| **Type of analysis** | **COVID-19** | **Control** | ***P* value** (**age**) |
| **Total no. of samples** | 16 (80 ± 10y) | 15 (68 ± 8y) | < 0.001 |
| Transversal sections | 16 (80 ± 10y) | 6 (72 ± 5y) | 0.086 |
| Transversal sections: terminal-pedicles area in cones | 8 (80 ± 10y) | 6 (70 ± 7y) | 0.360 |
| Transversal sections: axon width in cones | 10 (80 ± 10y) | 5 (68 ± 7y) | 0.1772 |
| Microglia quantification – flat-mount retinas | 7 (70 ± 10y) | 7 (71 ± 8y) | 0.877 |
| Microglia quantification on the vessels – flat-mount retinas | 5 (80 ± 10y) | 5 (66 ± 7y) | 0.1270 |
| Microglia quantification – transversal sections | 9 (80 ± 10y) | 5 (67 ± 7y) | 0.201 |
| TUNEL immunoassay | 10 (80 ± 10y) | 6 (72 ± 5y) | 0.323 |
| Spearman's correlation coefficient | 14 (80 ± 10y) | 6 (72 ± 5y) | 0.158 |

| **Table S2.** **Laboratory data of COVID-19 patients and score defined to each value.** | | | | | | | |
| --- | --- | --- | --- | --- | --- | --- | --- |
| **ID patient** | **Age**  **(years)** | **Sex** | **PaFI PaO_2_/FiO_2_** (**points**) | **SOFA** (**points**) | **DD ng/mL** (**points**) | **Total severity points** | **Stages of clinical severity** |
| CoV-1 | 84 | M | 243 (+2) | 5 (+2) | 1473 (+0) | 4 | 1 |
| CoV-2 | 84 | F | 232 (+2) | 4 (+2) | - | - | - |
| CoV-3 | 83 | M | 52 (+3) | 2 (+0) | 927 (+0) | 3 | 0 |
| CoV-4 | 64 | M | 50 (+3) | 5 (+2) | 1380 (+0) | 5 | 2 |
| CoV-5 | 71 | F | 254 (+2) | 2 (+0) | 841 (+0) | 2 | 0 |
| CoV-6 | 67 | M | 57 (+3) | 3 (+0) | 30477 (+2) | 5 | 2 |
| CoV-7 | 63 | M | 269 (+2) | 6 (+3) | 6277 (+0) | 5 | 2 |
| CoV-8 | 90 | M | 227 (+2) | 6 (+3) | 28393 (+2) | 7 | 2 |
| CoV-9 | 90 | M | 86 (+3) | 4 (+2) | 451 (+0) | 5 | 2 |
| CoV-10 | 88 | M | 519 (+0) | 0 (+0) | 4109 (+0) | 0 | 0 |
| CoV-11 | 91 | F | 220 (+2) | 5 (+2) | 3604 (+0) | 4 | 1 |
| CoV-12 | 92 | F | 343 (+1) | 9 (+3) | 602 (+0) | 4 | 1 |
| CoV-13 | 88 | F | 305 (+1) |  | - | - | - |
| CoV-14 | 98 | F | 495 (+0) | 10 (+3) | 745 (+0) | 3 | 0 |
| CoV-15 | 84 | M | 180 (+3) | 7 (+3) | 1915 (+0) | 6 | 2 |
| CoV-16 | 91 | F | 308 (+1) | 5 (+2) | < 200 (+0) | 3 | 0 |
| Values of PAFI, SOFA and DD for each patient are shown. Severity points are defined for each value (+0, +1, +2, +3). According to the total severity points, patients were classified in different stages of clinical severity: minor (0), moderate (1), or severe (2) disease. Normal values: PaFI > 300, DD = 0–232 ng/mL.  M = male; F = female; PaFI = partial pressure of arterial blood oxygen/fraction of inspired oxygen ratio; PaO_2_ = partial pressure of arterial blood oxygen; FiO_2_ = fraction of inspired oxygen; SOFA = sequential organ failure assessment; DD = D-dimer. | | | | | | | |

| **Table S3. Biomarkers, complications and treatments during hospital admission of COVID-19 patients.** | | | | | | | | | |
| --- | --- | --- | --- | --- | --- | --- | --- | --- | --- |
| **ID patient** | **Age**  **(years)** | **Sex** | **CRP** (**mg/L**) | **PCT** (**ng/mL**) | **IL-6** (**pg/mL**) | **Lymphocytes** (**10^9^/L**) | **LDH** (**IU/L**) | **Complications** | **Treatments during hospital admission** |
| CoV-1 | 84 | M | 16.0 | 16.62 | 45.00 | 0.5 | 774 | Sepsis, myocardial damage, acute renal failure | Dexamethasone |
| CoV-2 | 84 | F | 35.9 | 0.24 | - | 0.4 | 1039 | Acute renal failure | Dexamethasone |
| CoV-3 | 83 | M | 61.1 | 33.21 | - | 0.2 | 933 | No | Dexamethasone |
| CoV-4 | 64 | M | 4.4 | 0.07 | 12.82 | 0.2 | 758 | No | Tocilizumab, dexamethasone |
| CoV-5 | 71 | F | 38.3 | 16.61 | 14.14 | 0.2 | 1428 | No | Tocilizumab, dexamethasone |
| CoV-6 | 67 | M | 7.1 | 0.08 | - | 1.1 | - | Deep vein thrombosis, pulmonary thromboembolism | Dexamethasone |
| CoV-7 | 63 | M | 8.2 | 0.51 | 19.00 | 0.8 | 464 | Nosocomial bacterial superinfection, *de novo* atrial fibrillation, pulmonary thromboembolism | Dexamethasone |
| CoV-8 | 90 | M | 2.0 | 0.09 | 24.10 | 1.5 | 900 | No | Dexamethasone |
| CoV-9 | 90 | M | 9.7 | 4.12 | 11.99 | 0.4 | 1262 | No | No |
| CoV-10 | 88 | M | 21 | 49.78 | - | 0.8 | - | No | Dexamethasone |
| CoV-11 | 91 | F | 7.1 | 0.28 | - | 2.7 | - | No | Dexamethasone |
| CoV-12 | 92 | F | 17.1 | 0.08 | 679.00 | 0.4 | 1378 | No | Dexamethasone |
| CoV-13 | 88 | F | 38.9 | 0.3 | 16.48 | 0.3 | 1381 | No | Dexamethasone |
| CoV-14 | 98 | F | 23.5 | 1.56 | 50.16 | 0.2 | 1516 | Iatrogenic coagulation | No |
| CoV-15 | 84 | M | 13.5 | 0.11 | 117.60 | 0.4 | 1325 | Nosocomial bacterial superinfection, *de novo* atrial fibrillation | Dexamethasone |
| CoV-16 | 91 | F | 5.6 | 0.19 | 9.42 | 0.3 | 1646 | Heart failure and right upper limb hematoma | Dexamethasone |
| Laboratory data for different biomarkers are described. Normal values: CRP = 0–0.5 mg/L; PCT = 0–19.8 ng/mL; IL-6 = 4.631–5.740 pg/mL; Lymphocytes = 1.5–4.0 x10^9^; LDH = 208–378 IU/L.  M = male; F = female; CRP = C reactive protein; PCT = procalcitonin; IL-6 = interleukin-6; LDH = lactate dehydrogenase. | | | | | | | | | |

| **Cell type** | **Score** | **Control group**  (**Total sample size = 6**)  **n** (**%**) | **COVID-19 group**  (**Total sample size = 16**)  **n** (**%**) |
| --- | --- | --- | --- |
| Cone photoreceptor | Stage 0 Normal | 2 (33.3%) | 7 (43.8%) |
|  | Stage 1 Moderate | 3 (50%) | 3 (18.8%) |
|  | Stage 2 Severe | 1 (16.7%) | 6 (37.5%) |
| Müller cells | Stage 0 Normal | 3 (50%) | 3 (18.8%) |
|  | Stage 1 Moderate | 2 (33.3%) | 10 (62.5%) |
|  | Stage 2 Severe | 1 (16.7%) | 3 (18.8%) |
| Microglial | Stage 0 Normal | 4 (66.7%) | 1 (6.3%) |
|  | Stage 1 Moderate | 2 (33.3%) | 7 (43.8%) |
|  | Stage 2 Severe | 0 (0%) | 8 (50%) |

**Table S4. Frequency of observations in the immunohistochemistry images.**

| **ID patients** | **Age**  **(years)** | **Duration of illness** (**days** **from symptoms onset**) | **Severity** (**absolute values**) | **Severity score** | **PaFI values** | **Cones score** | **Müller cells score** | **Microglia activation score** | **Total retinal degeneration** |
| --- | --- | --- | --- | --- | --- | --- | --- | --- | --- |
| CoV-1 | 84 | 3 | 4 | 1 | 2 | 0 | 0 | 2 | 2 |
| CoV-2 | 84 | 2 | N/A | N/A | 2 | 0 | 1 | 2 | 3 |
| CoV-3 | 83 | 19 | 3 | 0 | 3 | 0 | 1 | 2 | 3 |
| CoV-4 | 64 | 32 | 5 | 2 | 3 | 2 | 2 | 1 | 5 |
| CoV-5 | 71 | 36 | 2 | 0 | 2 | 2 | 1 | 2 | 5 |
| CoV-6 | 67 | 16 | 5 | 2 | 3 | 1 | 1 | 2 | 4 |
| CoV-7 | 63 | 45 | 5 | 2 | 2 | 2 | 2 | 1 | 5 |
| CoV-8 | 90 | 15 | 7 | 2 | 2 | 1 | 1 | 2 | 4 |
| CoV-9 | 90 | 12 | 5 | 2 | 3 | 0 | 0 | 2 | 2 |
| CoV-10 | 88 | 23 | 0 | 0 | 0 | 0 | 1 | 1 | 2 |
| CoV-11 | 91 | 36 | 4 | 1 | 2 | 1 | 1 | 1 | 3 |
| CoV-12 | 92 | 13 | 4 | 1 | 1 | 2 | 1 | 1 | 4 |
| CoV-13 | 88 | 4 | N/A | N/A | 1 | 0 | 0 | 1 | 1 |
| CoV-14 | 98 | 16 | 3 | 0 | 0 | 0 | 1 | 1 | 2 |
| CoV-15 | 84 | 40 | 6 | 2 | 3 | 2 | 2 | 2 | 6 |
| CoV-16 | 91 | N/A | 3 | 0 | 1 | 2 | 1 | 0 | 3 |

**Table S5. Retinal and clinical data used in Spearman's correlation coefficient.**

The severity score was obtained considering D-dimer, PaFI and SOFA values. Some clinical data were incomplete for CoV-2 and CoV-13. Absolute values of the severity corresponded with different stages of clinical severity: minor (0), moderate (1), or severe (2) disease. Absolute values of PaFI levels were classified as: (+0) when PaFI was > 400, (+1) if PaFI values were comprised between 300-400, (+2) between 200-299, and (+3) if PaFI was < 200. Individual scores of cone photoreceptors, Müller cells and microglia were defined considering the stages of response to damage. Total retinal degeneration was obtained by the sum of the score of cones, Müller cells and microglia activation.

PaFI = partial pressure of arterial blood oxygen/fraction of inspired oxygen ratio; N/A = not available.

**Figure S1. Detection of cell death by TUNEL assay**. Representative pictures from vertical sections from control (a) and COVID-19 patients (b-d) after TUNEL staining. TO-PRO 3 iodide was used to visualize the nuclei (blue). Cell death was not detected in all studied human samples. Representative picture of vertical sections from rd10 mice retina at postnatal day 27 that were assayed in parallel as a positive control (e). High magnification of selected area (yellow square) showing the positive TUNEL reactivity at ONL (red arrows) in rd10 mouse indicating death of photoreceptors (f). ONL, outer nuclear layer; INL, inner nuclear layer; GCL, ganglion cell layer. Scale bars: 50 μm.


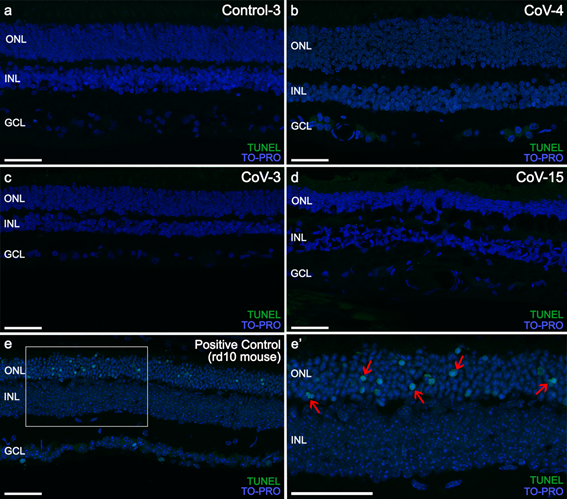


**Figure S2. Linear regression and Spearman's correlation coefficient** (***ro***) **of the different retinal cells**. The response to damage of cones and Müller cells showed a strong and significant correlation (a). No other significant correlations were observed for the different cells (b, c).


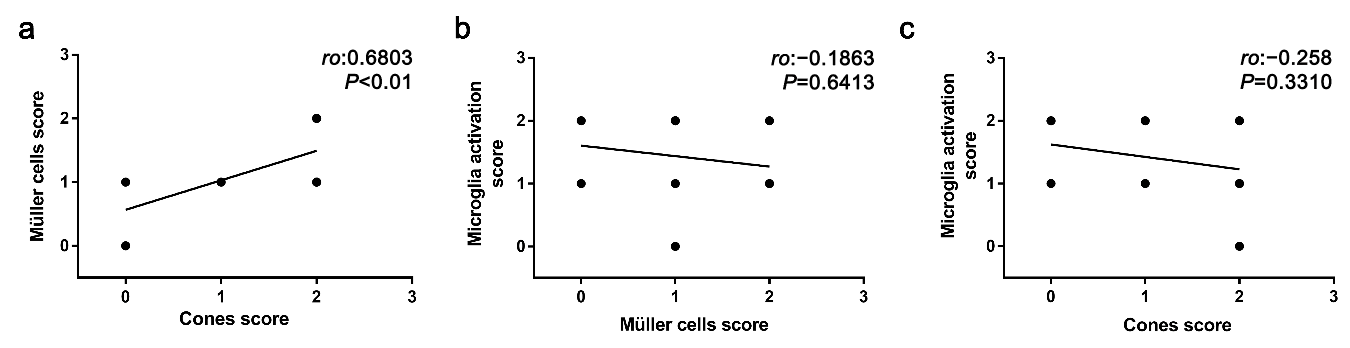


**Figure S3. Linear regression and Spearman's correlation coefficient** (***ro***) **between retinal cells and clinical data**. **a, b**) Stage of cone photoreceptors and Müller cells significantly correlated with the duration of the disease. **c**) No correlation was present between microglia activation and the duration of the illness. **d, e** The response of cones and Müller cells did not correlate with the PaFI values. **f** Microglia activation showed a strong correlation with the PaFI values. In this case, the higher PaFI values, the more compromised the patient's oxygen capacity and the higher the microglia response. Red squares pointed out the significant correlations.


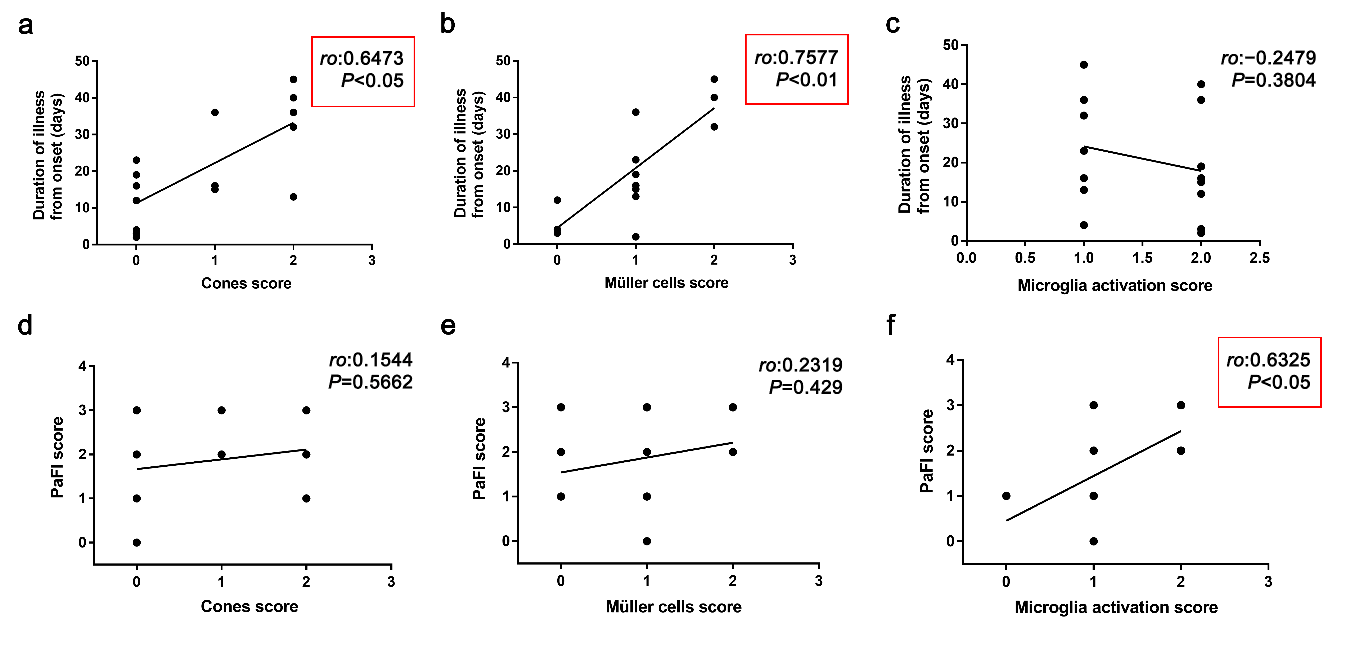

Supplement: Supplementary file 1 — Additional file 1: Table S1. Number of samples used for each analysis. Table S2. Laboratory data of COVID-19 patients and score defined to each value. Table S3. Biomarkers, complications and treatments during hospital admission of COVID-19 patients. Table S4. Frequency of observations in the immunohistochemistry images. Table S5. Retinal and clinical data used in Spearman's correlation coefficient. Fig S1. Detection of cell death by TUNEL assay. Fig S2. Linear regression and Spearman's correlation coefficient (ro) of the different retinal cells. Fig S3. Linear regression and Spearman's correlation coefficient (ro) between the retinal cells and clinical data. [file 40662_2023_329_MOESM1_ESM.docx]
